# Food Toxicity of Mycotoxin Citrinin and Molecular Mechanisms of Its Potential Toxicity Effects through the Implicated Targets Predicted by Computer-Aided Multidimensional Data Analysis

**DOI:** 10.3390/life13040880

**Published:** 2023-03-26

**Authors:** Seema Zargar, Tanveer A. Wani

**Affiliations:** 1 Department of Biochemistry, College of Science, King Saud University, Riyadh 11495, Saudi Arabia; 2 Department of Pharmaceutical Chemistry, College of Pharmacy, King Saud University, Riyadh 11451, Saudi Arabia

**Keywords:** fungi, citrinin, food toxicity, in-silico analysis, gene targets, protein targets

## Abstract

The mycotoxin citrinin, which can contaminate food, is a major global concern. Citrinin is regarded as an inevitable pollutant in foods and feed since fungi are widely present in the environment. To identify contentious toxicity and lessen its severity by understanding the targets of citrinin in the human body and the impacted biosynthetic pathways, we analyzed the production of citrinin from *Aspergillus flavus* and *Penicillium notatum* and used a thorough bioinformatics analysis to characterize the toxicity and predict genes and protein targets for it. The predicted median fatal dosage (LD_50_) for citrinin was 105 mg/kg weight, and it belonged to toxicity class 3 (toxic if swallowed). Citrinin was found to be well absorbed by human intestinal epithelium and was a Pgp nonsubstrate (permeability glycoprotein), which means that once it is absorbed, it cannot be pumped out, hence leading to bioconcentration or biomagnification in the human body. The main targets of toxicity were casp3, TNF, IL10, IL1B, BAG3, CCNB1, CCNE1, and CDC25A, and the biological pathways implicated were signal transduction involved in DNA damage checkpoints, cellular and chemical responses to oxidative stress, DNA damage response signal transduction by P53, stress-activated protein kinase signaling cascade, netrin–UNC5B signaling, PTEN gene regulation, and immune response. Citrinin was linked to neutrophilia, squamous cell carcinoma, Fanconi anemia, leukemia, hepatoblastoma, and fatty liver diseases. The transcription factors E2F1, HSF1, SIRT1, RELA, NFKB, JUN, and MYC were found to be responsible. When data mining was performed on citrinin targets, the top five functional descriptions were a cell’s response to an organic cyclic compound, the netrin–UNC5B signaling pathway, lipids and atherosclerosis, thyroid cancer, and controlling the transcription of the PTEN gene.

## 1. Introduction

Citrinin pollutants have been found in human biological fluids as well as agricultural commodities and feedstuffs such as cheese, sake, and soy sauce [1,2]. Citrinin has been identified in various foods such as meals colored with ***Monascus*** pigments, fermented sausages, wheat, corn, and rice [3,4,5,6]. Mycotoxins are poisonous substances that can grow on food, crops, or grains that have been stored before or after harvest. They are a category of hazardous, varied secondary metabolites produced by numerous fungi [4,7,8]. Mycotoxins are regarded as inevitable pollutants in foods and feed because fungi are common in the environment. The mycotoxin citrinin, one of the toxic secondary metabolites produced by various species of fungi, was first identified in the filamentous fungus *Penicillium citrinum* in 1931. Citrinin has been linked to human genotoxic, embryotoxic, teratogenic, carcinogenic, and mycotoxin nephropathy consequences. Citrinin is known to have a variety of bioactivities in vitro, including antibacterial, antifungal, and possible anticancer and neuroprotective properties, in addition to its toxic effects. Despite having antibiotic properties, it was never used as an antibiotic because it showed toxic effects on mammals, particularly nephrotoxicity [8]. Citrinin is a solid polyketide that is yellow and has the chemical formula C_13_H_14_O_5_. Its IUPAC name is (3R,4S)-6-hydroxy-3,4,5-trimethyl-8-oxo-3,4-dihydroisochromene-7-carboxylic acid. Citrinin pollutants have been found in human biological fluids as well as agricultural commodities and feedstuffs such as cheese, sake, and soy sauce [7,8]. Citrinin has been identified in meals colored with *Monascus* pigments, fermented sausages, wheat, corn, and rice [4]. Numerous human cell lines and animal species have been used to study the toxicity of citrinin, and in tissues, kidney injury and changes in the metabolism of the liver have also been reported [9,10,11,12]. The DNA adduct C-C8dG-OTA was formed as a result of the co-exposure to citrinin and ochratoxin A [13]. Citrinin’s mechanism of action on mitochondrial metabolism has been elucidated, and it has been observed that in the liver and kidney mitochondria of rats, citrinin inhibits several enzymes, including malate dehydrogenase and glutamate dehydrogenase, as well as the ATP synthase complex involved in the respiratory chain [14]. Through the transcription factors Skn7 and Yap1, citrinin has been shown to boost a dose-dependent enhanced expression of GRE2 or SOD2 promoters with stress-sensitive promoter characteristics [4,15,16]. Citrinin is known to have a wide range of bioactivities in addition to its toxic effects, including antibacterial, antifungal, and possible anticancer and neuroprotective properties in vitro [17]. Over the dose and test systems employed, there is still debate regarding the genotoxic and mutagenic potentials of citrinin. Hot water, sodium carbonate, sodium hydroxide (aqueous), sodium acetate, and polar organic solvents including methanol, ethanol, and acetonitrile are among the media in which citrinin is soluble. Depending on the type of solvent employed for dissolution, UV light can be most effectively absorbed between 250 and 321 nm [18]. Although there are few reports on its biosynthesis and more research is required, it is predicted that the majority of secondary metabolites, including pigments, monacolin K, and citrinin, share a common biochemical pathway in branching the biosynthetic pathway produced from acetyl CoA and malonyl-CoA. According to the literature, the ctnA, PksCT, orf1, 3, 4, and 5 genes, which encode a regulator polyketide synthetase, an aldehyde dehydrogenase, an oxygenase, an oxidoreductase, and a membrane transporter, are each involved in the biosynthesis process [19,20,21]. The toxin affects all the main organs, including the bone marrow, liver, kidney, and mitochondrial respiratory chain [22]. The negative consequences are thought to be caused by altered enzymatic antioxidative responses and the effects of oxidative stress. Altering the gene expression of jun B and tbx2a in Zebrafish causes decreased blood flow and heartbeat, as well as male infertility [23]. Citrinin accelerated apoptotic processes, reduced total cell counts, and interfered with oocyte maturation, fertilization, and embryonic development in mouse blastocysts [24]. Given that citrinin is a common food contaminant found in human food all over the world, it is logical to assume that humans are exposed to it far more frequently than is usually believed. Due to its poisonous, mutagenic, and carcinogenic characteristics, citrinin contamination is one of the biggest hazards to food safety and human health. As a result, more studies are needed to cope with the contamination of feed and food commodities with citrinin, whereas there is relatively little knowledge regarding this contamination’s toxicological effects. Large sample sizes, low costs, and the capacity to conduct extensive functional analyses and genomics research through computer-aided multi-dimensional data analysis are the distinguishing features of the approaches currently available. It may be possible to identify contentious toxicity and lessen its severity by understanding the toxicity targets of citrinin in the human body and the impacted biosynthetic pathways by these in silico analyses. In this study, we used thin-layer chromatography (TLC) to analyze the production of citrinin from *Aspergillus flavus* and *Penicillium notatum*. We then used a thorough bioinformatics analysis and toxicity characterization to determine the disease-causing targets for citrinin. To further elucidate citrinin’s potential role in human toxicity, its cheminformatic aspects were studied. Computer models have been promoted as a viable alternative to experimental methods when knowledge about compounds is limited.

## 2. Material and Methods

### 2.1. Fungal Culture and Analysis of Citrinin Production

Potato dextrose agar (PDA) (39 g) was dissolved in distilled water to make a one-liter solution (1L). After autoclaving for 15–20 min at 121 °C, 50 mg/L streptomycin was added after cooling, and 20–25 mL portions were dispensed into sterile 15 × 100 mm Petri dishes. *Penicillium notatum* and *Aspergillus flavus* fungal cultures were maintained and grown on Petri dishes at 25 °C for 10 days. The spores were suspended and harvested by adding 10 mL of sterilized distilled water to each plate. The spore suspension thus obtained was filtered using cheesecloth. The filtered biomass was then dried at 40 °C for 24 h, and the dry weight of mycelium was determined. Filtrates were stored at 4 °C for carrying out citrinin extraction, and the weight of the biomass was recorded. Each experiment was performed in duplicate. To validate the production of citrinin, thin-layer chromatography (TLC) was performed on the acquired culture filtrates. Citrinin was extracted three times with chloroform (1:1 *v*/*v*) added to the filtered solution for the TLC. The lowest layer of the separated mixture was collected. At 40 °C, the fractions were dried in a dry oven. The synthesis of citrinin was verified by TLC under UV light after dilution of citrinin extracts with 2 mL of chloroform. Briefly, sample extracts and a standard (containing 0.5 g/mL) from Sigma Chemical Co. were applied to TLC plates (St. Louis, MO, USA). In glass tanks lined with aluminum foil, TLC plates were produced in a solvent composed of toluene, ethyl acetate, and formic acid (6:4:0.5 *v*/*v*). After development, plates were dried for 30 min to one hour, and when viewed under wavelength (365 nm), a bright yellow spot corresponding to citrinin was seen. Each experiment involved duplicates. 

### 2.2. Toxicity Prediction by ProTox-II

For the prediction of various toxicity endpoints of citrinin (PubChem CID: 54680783), such as acute toxicity, hepatotoxicity, cytotoxicity, carcinogenicity, mutagenicity, immunotoxicity, adverse outcome pathways (Tox21), and toxicity targets, ProTox-II (https://tox-new.charite.de/protox_II/, accessed on 15 January 2023) was used. ProTox-II incorporates molecular similarity, pharmacophores, fragment propensities, and machine-learning models. The predictive models are based on data from both in vivo instances and in vitro assays, such as the Tox21 assay, the Ames bacterial mutation assay, the hepG2 cytotoxicity assay, and the immunotoxicity assay (e.g., carcinogenicity, hepatotoxicity). The models have performed well and have been validated on separate external sets. The predicted median fatal dosage (LD_50_) was obtained in mg/kg weight along with the toxicity class, and the prediction accuracy [25]. 

### 2.3. Toxicity Radar Targets of Citrinin

Fast yet robust predictive models for physicochemical properties such as BOILED-Egg, iLOGP, and bioavailability radar were generated from SwissADME (http://www.swissadme.ch/, accessed on 15 January 2023) [26], with an emphasis on predicting the toxicity profiles of citrinin. The Swiss target prediction tool was used to predict the genes and protein targets affected directly or indirectly by toxicity [27]. SwissADME uses canonical SMILES of compounds to analyze the target binding. The BOILED-Egg model was used to predict the penetration of the blood–brain barrier (BBB) and passive human gastrointestinal absorption (HIA) [28]. Furthermore, the target genes that are implicated in citrinin toxicity in several species were predicted using the Comparative Toxicogenomics Database (http://ctdbase.org/, accessed on 15 January 2023). The SMILES for citrinin were obtained from (PubChem CID: 54680783). The database contains data on multiple genes and proteins that differ between various species and respond to environmental toxins such as citrinin. Comparative Toxicogenomics Database (CTD) has significantly broadened its scope to accurately reflect a trio of hand-selected chemical–gene, chemical–disease, and gene–disease connections [29].

### 2.4. Gene Interaction Networks and Pathway Enrichment Analysis

The homosapiens option was used to analyze the target genes implicated in SwissADME, a comparative toxicogenomics database, and the ProTox-II tool for citrinin in GeneMANIA (https://genemania.org/, accessed on 15 January 2023) to analyze the gene sets interacting with one another for physical interaction, genetic interaction, co-expression, co-localization, shared protein domains, or predicted interactions. A real-time multiple association network integration approach was used to forecast gene function. GeneMANIA was used to discover any additional genes connected to a group of input genes. GeneMANIA creates gene networks by scoring nodes using functional genomic data and label propagation. The network can then be subjected to any analytic feature available in Cytoscape or Metascape. Gene function networks were shown by using colors based on the network nodes by function (GO annotation), and a color key was given in the legend [30,31]. To better understand the effect of citrinin, the collected gene list was evaluated through enrichment analysis using Metascape (https://metascape.org/gp/index.html#/main/step1, accessed on 15 January 2023) to find biological pathways and protein–protein interaction enrichments. It uses a variety of functional databases, such as GO, KEGG, and UniProt, to analyze data from a variety of other species in addition to data from the literature. It also evaluates not just a single dataset but also many gene sets simultaneously. Gene identities (Entrez Gene IDs) were automatically converted into human Entrez Gene IDs by Metascape using the EggNOG and Homologene databases, and all the transcription factors and pathways implicated with the appropriate *p* values were reported. Hypergeometric accumulative distributions recorded *p*-values to collect datasets and enrichment factors [32].

## 3. Results and Discussion

**Figure 1:**
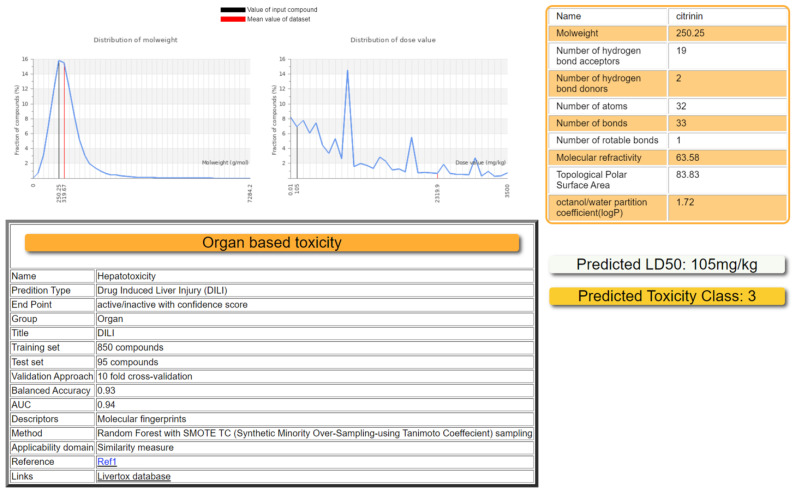
Toxicity characteristics of citrinin. The mean molecular weight (MW) of the endogenous compound is indicated as a red line, whereas the MW of citrinin is indicated as a black line. The distribution of LD_50_ values in the endogenous dataset is shown in red, and the predicted median lethal dose of citrinin is shown in black.

Citrinin is a secondary metabolite made by some fungal strains, particularly *Aspergillus*, *Penicillium,* and *Monascus* species. This natural poison is extremely prevalent and is mostly found in soil, rotting vegetation, and food storage systems. Due to its poisonous, mutagenic, and carcinogenic characteristics, citrinin contamination is one of the most significant risks to food safety and human health. *Penicillium notatum* and *Aspergillus flavus* were grown on PDA cultures. TLC analysis confirmed citrinin production by both studied strains. Further, citrinin’s potential toxicity in humans was elucidated by an in silico approach based on gene and protein toxicity targets and implicated transcription factors. This study is valuable since it can help to lessen the severity of citrinin toxicity by understanding its binding to gene and protein targets in the human body and the impacted biosynthetic pathways. The ProTox-II predicted median fatal dosage (LD50) for citrinin was 105 mg/kg weight (Figure 1). The ProTox-II webpage accepts a two-dimensional chemical structure as input and provides a report on the chemical’s potential toxicity profile for 33 models with confidence scores, an overall toxicity radar chart, and the three similar compounds with the highest known acute toxicity. The predictions were made easier for both the acute toxicity and toxicity targets. The mean molecular weight (MW) of the dataset was indicated as a red line, whereas the MW of the input compound (citrinin) was indicated as black (Figure 1). The mean molecular weight and dose distribution of the citrinin can be used for animal studies as the software compares these values obtained for citrinin with similar compounds in the database, considering 33 other models. The ability of in silico approaches to provide major benefits to both regulatory needs and requirements for risk assessments, as well as the pharmaceutical sector’s ability to analyze the safety profile of citrinin, has been made feasible by advancements in the field of computational research [25].

Further, the prediction accuracy and the known rodent oral toxicity value with 10-fold cross-validation were obtained for liver toxicity. The liver toxicity targets were displayed with the projected toxicity information and the probability score of the predicted outcomes (Table 1).

According to Table 1, citrinin is mutagenic, carcinogenic, and hepatotoxic and alters the expression of targets such as the aryl hydrocarbon receptor (AhR), the androgen receptor (AR), and aromatase, a member of the cytochrome P450 superfamily. The aryl hydrocarbon receptor is a transcription factor that is normally inactive, but once it binds to xenobiotics, it alters the gene expression of several enzymes important for differentiation. All three targets together alter the expression of genes involved in differentiation, androgen production, and cancer incidence. Previously, it was shown that mycotoxins activated AhR, which later influenced immunogenicity and epithelial organization during stress and inflammation [33] in an aromatase-dependent manner [34].

**Figure 2:**
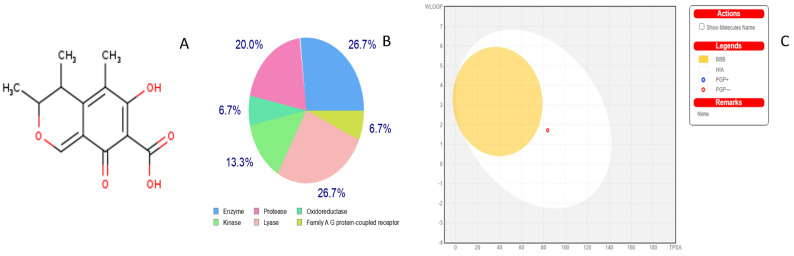
(**A**) Citrinin structure was adapted from Swiss ADME’s Marvin J S tool; (**B**) target classes binding to citrinin was adapted from Swiss ADME’s target prediction tool; (**C**) boiled egg demonstration shows that citrinin is expected to be well absorbed by passive gastrointestinal absorption and is brain impermeant. The presence of citrinin (red dot), in the egg white, demonstrates it as Pgp nonsubstrate rather than being pumped out if consumed, and, hence, may lead to bioconcentration or biomagnification.

It is common knowledge that hazardous doses are frequently expressed as LD50 values in mg/kg body weight, where LD50 denotes the median lethal dose following exposure to a substance. According to the GHS, there are six categories of toxicity: Class I, which is fatal if swallowed (LD50 5); Class II, which is also fatal if swallowed (LD50 ≤ 50), Class III, which is toxic if swallowed (LD50 ≤ 300), Class IV, which is harmful if swallowed (LD50 ≤ 2000), Class V, which may be harmful if swallowed (LD50 5000), and Class VI which is nontoxic (LD50 > 5000). In our preliminary investigation, it was estimated (93–94% accuracy) that citrinin should be classified in category III [35]. Consumption of citrinin-contaminated food and feed by both humans and animals has led to serious health concerns across the globe. The toxin could enter the food chain by contaminating food and feed at any stage of agricultural practice and in pre/post-harvest conditions. Due to the nephrotoxic and genotoxic nature of citrinin, the health of both humans and animals is at greater risk; therefore, target prediction in the human body for citrinin needs much attention [4,36]. The structure of citrinin was obtained (Figure 2A), and human targets were predicted by SwissADME (Figure 2B). The BOILED-Egg model demonstrated an easy interpretation and effective molecular design translation with a wide chemical range of prediction as to whether citrinin is only capable of passive penetration via the stomach, intestines, or blood–brain barrier (BBB). The Pgp substrate prediction, which is the most significant active efflux mechanism involved in those biological barriers, was acquired for the graphical output within SwissADME [37,38]. By using color coding, a boiled egg can provide information about passive absorption, passive brain access (inside/outside the yolk), and active efflux from the central nervous system (CNS) or gastrointestinal lumen. Blue dots indicate Pgp substrates (PGP+), while red dots indicate Pgp nonsubstrates (PGP) in the function of the position of the molecules in the WLOGP versus TPSA referential. Citrinin is predicted to be well absorbed and access the brain (in the egg white) and Pgp nonsubstrate (red dot), and not pumped out of the brain (Figure 2C). Furthermore, based on the excellent solubility values, we hypothesize that citrinin could be effectively absorbed orally after food consumption in the small intestine. Contrary to our study, some reports have concluded that citrinin crosses the BBB model in vitro faster than other mycotoxins [35,39].

**Figure 3:**
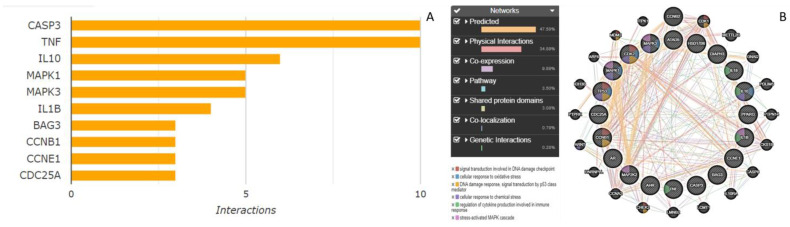
A network of protein–protein interactions and pathways implicated through (**A**) CTD and (**B**) GeneMANIA. In total, 41.5% of genes showed physical interactions and 23.54% were coexpressed and 28.67% were predicted to interact with each other. Caspase-3 and TNF showed 10 interactions and 5 interactions were shown by IL-10 and MAP kinases.

We mined functionally related genes from SwissADME, ProTox-II, and the Comparative Toxicogenomics Database (CTD) and created a network of protein–protein interactions through the GeneMANIA database. According to data in the plasma proteomic signature of healthy adults, functional enrichment revealed that the hub genes and functionally related genes that were mostly implicated in the CTD database were casp3, TNF, IL10, IL1B, BAG3, CCNB1, CCNE1, and CDC25A (Figure 3A). Benzo[a]pyrene (B(a)P) is a ubiquitous environmental contaminant and a toxic carcinogen that induces subchronic neural toxicity through the enhancement of Bcl-2, C-myc, Ki-67 oncogenes, and p53, Bax, and Caspase-3 proapoptotic gene expression [40]. Citrinin, also a ubiquitous environmental contaminant, affects the expression of casp3, TNF, IL10, IL1B, BAG3, CCNB1, CCNE1, and CDC25A genes in a similar fashion to B(a)P and showed carcinogenicity on analysis using ProTox-II. A previous study reported induction of apoptosis in HL-60 cells by stimulating cytochrome c release followed by activation of multiple caspases with citrinin exposure [41]. Another study reported that low exposure doses of citrinin (10 μg/mL) and gliotoxin (100 ng/mL) inhibited IL-10 and led to an increased risk of an inflammatory response with relative overproduction of TNF-α and IL-6 [42]. Furthermore, our study reports targets that may be implicated with citrinin exposures and, hence, need to be explored experimentally in vitro in animal cells or in vivo in animal models. Over the past few decades, there have been numerous significant advancements in high-throughput screening methods that have substantially influenced the pathophysiology of numerous diseases [43]. To comprehend the biological mechanisms and pathways underlying these risk signatures, we carried out gene set enrichment analysis to better understand how citrinin shows toxicity and what interactions are involved. The collected gene list was analyzed through enrichment analysis using GeneMANIA to find biological pathways and protein–protein interaction enrichments. GeneMANIA uses a variety of trustworthy functional databases, such as GO, KEGG, and UniProt, to analyze data from a variety of other species in addition to data from humans—not just a single dataset, but also many gene sets simultaneously. The biological pathways identified were signal transduction involved in the DNA damage checkpoint, cellular response to oxidative stress, chemical response to oxidative stress, DNA damage response signal transduction by P53, stress-activated protein kinase signaling cascade, and immune response (Figure 3B). Citrinin toxicity may be prevented and treated by targeting these genes or proteins; hence, the disease outcome will depend less on exposures, as they may be the key targets for biosurfactants. A study concluded that biosurfactants can be produced against the obtained targets that may possess promising antilisterial properties. In this study, we laid the groundwork for the identification of novel targets for citrinin exposure, which can be further explored to alleviate citrinin toxicity [43]. The recent developments in computational chemistry, theoretical modeling, bioinformatics of functional materials, drug design, and toxicity predictions of compounds give the most significant predictions of effects on biological and chemical systems using computational approaches.

**Figure 4:**
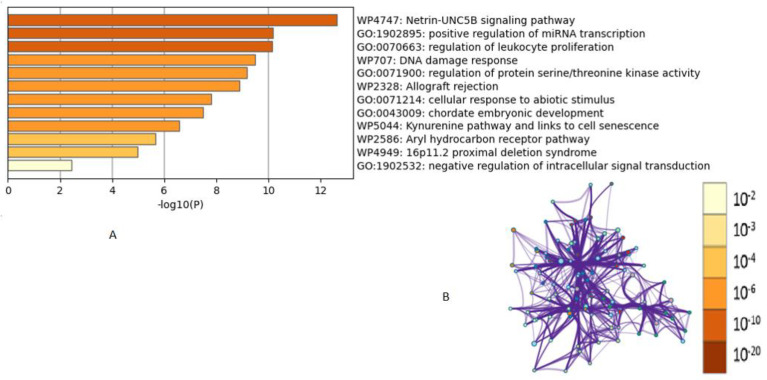
Statistically significant enriched terms are hierarchically clustered. (**A**) Enrichment analysis clustered into a tree based on Kappa-statistical similarities among their gene memberships; and (**B**) network clusters converted into a network layout. Terms showing a similarity score > 0.3 are linked by an edge in the network and nodes of the same color belong to the same cluster. The darker the color, the more statistically significant the node.

Our results are consistent with the findings from previous studies on cell lines reporting that the inhibition of the JNK signaling pathway improves cell viability in response to oxidative stress [44]. To fully comprehend the relationship between genes and disorders, Metascape was utilized to simultaneously assess important genes for pathway enrichment and functional annotation of genes. Metascape using the EggNOG and Homologene databases gave a mechanism of all the transcription factors and pathways implicated with the appropriate *p*-values. Hypergeometric accumulative distributions recorded *p*-values to collect datasets and enrichment factors. Citrinin targets the DNA damage response, positive regulation of miRNA transcription, modulation of the netrin–UNC5B signaling pathway, leukocyte proliferation regulation, etc. Multiple malignancies have been linked to UNC5B signaling, which boosts cell survival when its ligand netrin-1 is present and promotes cell death when it is not. Additionally, it has been noted that blocking the ligand reduces tumor invasiveness and angiogenesis [45]. Citrinin was significantly associated with neutrophilia, squamous cell carcinoma, Fanconi anemia, leukemia, hepatoblastoma, and fatty liver diseases. According to the Kappa-statistical similarity between the gene memberships of significant pathways, a tree was hierarchically clustered (Figure 4A), and a threshold of 0.3 kappa was used to divide the tree into word clusters. A subset of representative terms from the full cluster was converted into a network layout where each term was represented by a circle node with a size proportional to the number of input genes that fall under this term. The nodes of the same color showed the same cluster identity, and the thickness of the edge represented the similarity score (Figure 4B).

**Figure 5:**
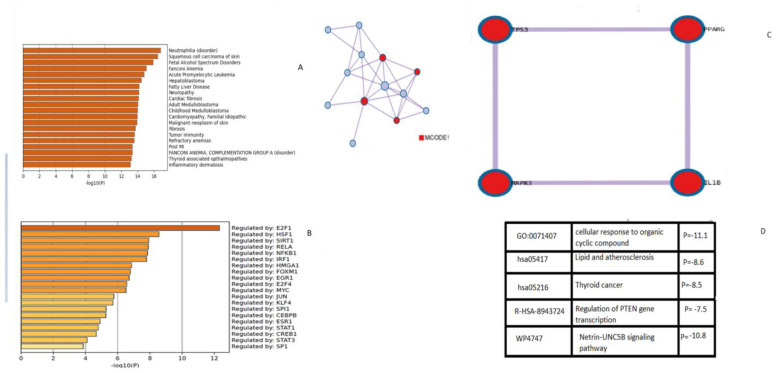
GO enrichment analysis with biological meanings. (**A**) The predicted disease outcomes by citrinin exposure; (**B**) the transcription factors implicated in disease outcome; (**C**) the MCODE algorithm identifying densely connected proteins; (**D**) the top five best *p*-value terms were retained and disease outcomes with these top significant *p*-values are shown in the table.

The molecular complex detection (MCODE) method was used to identify intricately connected network components in networks with 3 to 500 proteins. Circle plots are used to display their overlap and shared term relationships. The five terms with the greatest *p*-values from the various route and process enrichment experiments were retained as the functional descriptions of the various MCODE components (Figure 5). 

The top five clusters and their representative enriched terms are presented in the table with functional descriptions obtained with citrinin targets. These were a cellular response to an organic cyclic compound, the netrin–UNC5B signaling pathway, lipids and atherosclerosis, thyroid cancer, and regulation of PTEN gene transcription. Citrinin was found to change a state or activity in a cell in terms of movement, secretion, enzyme production, gene expression, etc. as a result of an organic cyclic compound stimulus. Citrinin’s mechanism of toxicity exposure has been elucidated, and it has been observed that citrinin targets several genes and proteins that are implicated in the diseases mentioned, and the transcription factors regulating these genes have also been elucidated. Previous studies reported enzymes including malate dehydrogenase and glutamate dehydrogenase as well as the ATP synthase complex involved in the respiratory chain implicated with citrinin exposures [14]. Other studies reported the transcription factors Skn7 and Yap1 regulating the expressions implicated in citrinin toxicity. Citrinin has been reported to boost a dose-dependently enhanced expression of GRE2 or SOD2 promoters with stress-sensitive promoter characteristics. Citrinin exposure may induce apoptosis. The transcription of the PTEN gene is regulated at multiple levels. PTEN was required for p53-mediated apoptosis in immortalized mouse embryonic fibroblasts [46]. It was proposed that citrinin shows its toxicity by positively regulating MAP kinase activity, positive regulation of miRNA transcription, protein kinase B signaling, DNA damage response signal transduction by P53, stress-activated protein kinase signaling cascade, netrin–UNC5B signaling, as well as an immune response. Citrinin was significantly associated with neutrophilia, squamous cell carcinoma, Fanconi anemia, leukemia, hepatoblastoma, and fatty liver diseases, and the transcription factors implicated were E2F1, HSF1, SIRT1, RELA, NFKB, JUN, and MYC. The top five functional descriptions obtained with data mining on citrinin targets were a cellular response to an organic cyclic compound, the netrin–UNC5B signaling pathway, lipids and atherosclerosis, thyroid cancer, and regulation of PTEN gene transcription. In mammalian cells, the stress-activated protein kinases (SAPKs), including RK/p38/CSBP kinase, and c-Jun N-terminal kinase (JNK), all together play very important roles in response to environmental stresses [46]. Upon activation, these stress-related protein kinases phosphorylate and activate the transcription factors ATF2, c-Jun, and Elk-1, to respond to the stress [47]. Previously, a possible mechanism was hypothesized for quercetin protection against CCL4 toxicity in the rat brain [48]. The NF-κB pathway has long been recognized as a classic proinflammatory signaling pathway. Increased NF-B activity indirectly encourages the development of neutrophil extracellular traps (NET), one of neutrophils’ antimicrobial defense mechanisms [49]. It has been demonstrated that Sirtuin 1 (SIRT1) binds to HSF1 and controls the acetylation status of HSF1 to act as a regulator of HSF1 DNA-binding activity [50]. Taken together, our results showed that citrinin toxicity mechanisms are attenuated via attenuation of the top five stress-related pathways in a protein kinase-, NF-κB-, and SIRT1-dependent manner, and that RELA, JUN, and MYC are involved in this process. To provide the framework for future research and development, this study examined the pharmacological mechanisms underlying citrinin’s toxicological action. To provide the framework for future research and development, this study examined the pharmacological mechanisms underlying citrinin’s toxicological action.

## 4. Conclusions

This study investigated the pharmacological mechanisms underlying the toxicological activity of citrinin to provide a platform for future research and development. The lethal dose of citrinin has been predicted (Figure 1). Citrinin, a potential carcinogen, demonstrates its harmful effects through upregulating MAP kinase activity, miRNA transcription, protein kinase B signaling, DNA damage response signal transduction by P53, a stress-activated protein kinase signaling cascade, netrin–UNC5B signaling, and an immunological response (Figure 4 and Figure 5). The transcription factors involved were E2F1, HSF1, SIRT1, RELA, NFKB, JUN, and MYC (Figure 5B). Citrinin was substantially related to neutrophilia, squamous cell carcinoma, Fanconi anemia, leukemia, hepatoblastoma, and fatty liver disorders (Figure 5A). The netrin–UNC5B signaling pathway, lipids and atherosclerosis, thyroid cancer, and modulation of PTEN gene transcription were the top five functional descriptions discovered using data mining on citrinin targets (Figure 5D). When combined, our results will pave the way for additional in vivo and in vitro studies on citrinin to identify contentious toxicity and lessen its severity by understanding the targets of citrinin in the human body and the impacted biosynthetic pathways. Additional computer simulations could be performed in the future, including quantum-mechanical calculations of citrinin’s chemical reactivity as well as molecular dynamics simulations of citrinin binding coupled with free-energy calculations.

## Figures and Tables

**Table 1:** Toxicity Prediction model with probability scores. A probability score of more than 0.8 is considered positive and less than it is possibly positive. The values less than 0.5 were considered negative and excluded.

Effect	Target	Probability
Organ toxicity	Hepatotoxicity	0.7
Toxicity endpoints	Carcinogenicity	0.56
Toxicity endpoints	Immunogenicity	0.69
Toxicity endpoints	Mutagenicity	0.85
Toxicity endpoints	Cytotoxicity	0.77
Tox21-nuclear receptor signaling pathways	Aryl hydrocarbon receptor (AhR)	0.85
Tox21-nuclear receptor signaling pathways	Androgen receptor (AR)	0.93
Tox21-nuclear receptor signaling pathways	Androgen receptor ligand-binding domain (AR-LBD)	0.96
Tox21-nuclear receptor signaling pathways	Aromatase	0.75
Tox21-nuclear receptor signaling pathways	Estrogen receptor alpha (ER)	0.75
Tox21-nuclear receptor signaling pathways	Estrogen receptor ligand-binding domain (ER-LBD)	0.91
Tox21-nuclear receptor signaling pathways	Peroxisome proliferator-activated receptor gamma (PPAR-Gamma)	0.94
Tox21-stress response pathways	Nuclear factor (erythroid-derived 2)-like 2/antioxidant response element (nrf2/ARE)	0.85
Tox21-stress response pathways	Heat shock factor response element (HSE)	0.85
Tox21-stress response pathways	Mitochondrial membrane potential (MMP)	0.61
Tox21-stress response pathways	Phosphoprotein (tumor suppressor) p53	0.83
Tox21-stress response pathways	ATPase family AAA domain-containing protein 5 (ATAD5)	0.9

## Data Availability

Data will be available on request to the corresponding author.
